# Comparative efficacy of different drugs in acute heart failure with renal dysfunction: a systematic review and network meta-analysis

**DOI:** 10.3389/fcvm.2024.1444068

**Published:** 2025-01-14

**Authors:** Qianyu Lv, Qian Wu, Yingtian Yang, Lanlan Li, Xuejiao Ye, Shihan Wang, Yanfei Lv, Manshi Wang, Yushan Li

**Affiliations:** ^1^ Guang’anmen Hospital, China Academy of Chinese Medical Sciences, Beijing, China; ^2^ School of Management, Fudan University, Shanghai, China; ^3^ Emergency Department, Guangwai Hospital, Beijing, China; ^4^ Institute of Traditional Chinese Medicine, Beijing University of Chinese Medicine, Beijing, China

**Keywords:** acute heart failure, renal insufficiency, diuretics, high dose of diuretics, network meta-analysis

## Abstract

**Objective:**

This network meta-analysis was to compare the efficacy of different drugs on cardiac function, renal function, and clinical outcomes in patients with acute heart failure (AHF) accompanied by renal dysfunction.

**Methods:**

PubMed, EMBASE, Cochrane Library, and Web of Science were searched to screen all clinical trials of AHF between January 1st 2001 and March 31th 2024. The primary outcome measures were N-terminal pro-B type natriuretic peptide (NT-proBNP), B-type natriuretic peptide (BNP), glomerular filtration rate (GFR), blood urea nitrogen, serum creatinine, all-cause mortality within 60 days, and cardiovascular mortality.

**Results:**

After screening 30,697 citations, 13 studies (21,745 patients) were included, and drugs including nesiritide, dopamine, tolvaptan, levosimendan, dobutamine, furosemide, and spirolactone, and high dose of diuretics (HDD, furosemide, and spirolactone) were estimated. The results indicated that HDD had the best efficacy in reducing NT-proBNP levels. In detail, HDD notably reduced NT-proBNP levels compared to conventional treatment or placebo (PLC) [MD = −950.24; 95% CrI (−1,832.21, −64.12)]. Levosimendan significantly increased GFR levels compared to PLC [MD = 14.46; 95% CrI (3.88, 25.97)] and tolvaptan [MD = 13.83; 95% CrI (2.31, 25.33)]. No significant difference was found in 60-day all-cause mortality and cardiovascular mortality across drugs.

**Conclusion:**

HDD showed the best efficacy in reducing NT-proBNP levels compared with dopamine and nesiritide, and levosimendan could significantly improve GFR levels, with no marked difference in the effect of various drugs on 60-day all-cause mortality. Hence, HDD and levosimendan may be optimal agents in the treatment of AHF with renal dysfunction.

**Systematic Review Registration:**

PROSPERO, identifier (CRD42023454616).

## 1 Introduction

Acute heart failure (AHF) is one of the most common acute cardiovascular diseases that appears or deteriorates rapidly after cardiac dysfunction, mainly characterized by dyspnea, pulmonary edema, pulmonary congestion, rapid heart rate, and elevated plasma natriuretic peptide levels (1). In AHF, myocardial contractility is reduced, resulting in a decrease in cardiac output (2) and renal blood perfusion insufficiency. Therefore, renal dysfunction is also a common complication of AHF.

The relationship between heart failure (HF) and renal function is complex since the disease itself, compensatory mechanisms, congestion, and medication can all impair renal function (3). A decline in renal function often leads to forced drug withdrawal (4) which accelerates HF progression and predetermines higher mortality and hospitalization rates in AHF patients (5, 6). One study (7) reported that 27%–45% of AHF patients experienced worsening renal function (WRF). Compared to those without, the mortality rate increased in such patients by 1.72–6.5 times, and the incidence of complications such as myocardial infarction or shock increased by 1.1 times. A meta-analysis also showed that renal protection therapy can greatly improve outcomes of HF patients (3). AHF patients combined with renal dysfunction are at risk for hyperemia and WRF, both of which can lead to unfavorable outcomes. Therefore, it is necessary to relieve congestion while protecting renal function when treating AHF (8–10).

Guideline-directed medical therapy significantly impacts mortality and morbidity in HF patients (11, 12), including diuretics, natriuretic peptides, positive inotropic drugs, vasoactive agents, β-receptor blockers, and sodium-glucose transporter 2 inhibitors (SGLT2i). Among them, diuretics are often considered the basis of HF treatment. AHF patients with renal dysfunction generally do not respond well to traditional diuretics and even develop diuretic resistance, which aggravates renal damage (13, 14). Although some emerging drugs such as nesiritide and tolvaptan have been approved for AHF treatment, their clinical efficacy on renal function remains controversial (15, 16). It is therefore necessary to compare the efficacy and safety of these drugs on cardiovascular outcomes and renal function in AHF patients with renal dysfunction to find a better therapeutic regimen.

However, to date, the efficacy of different agents in AHF patients with renal dysfunction has not been identified. A network meta-analysis (NMA) can simultaneously compare the effects of multiple interventions because of the lack of direct comparisons between these drugs (17). In addition, the NMA can rank interventions based on various outcomes. Thus, this study aims to compare the efficacy of different drugs on cardiac function, renal function, and clinical outcomes in patients with acute heart failure (AHF) combined with renal dysfunction, providing some references for clinicians to make evidence-based decisions.

## 2 Methods

### 2.1 Study design and registration

We designed and wrote this paper following the Preferred Reporting Items for Systematic Reviews and Meta-Analyses (PRISMA) 2020 statement (18), and registered the protocol with the PROSPERO (CRD42023454616).

### 2.2 Eligibility criteria

Articles were included according to the following criteria: (1) Patients diagnosed with AHF combined with renal dysfunction of any age. The diagnosis of AHF was based on the Framingham criteria (19), accompanied by at least one of the following symptoms: pulmonary congestion, peripheral edema, pleural effusion, jugular venous distension, or orthopnea. Renal dysfunction was regarded as a glomerular filtration rate (GFR) of 15 to 60 ml/min per 1.73 m^2^ estimated by the Modification of Diet in Renal Disease equation (20, 21). (2) Patients in the intervention group took at least one type of the guidance-recommended medications for AHF, including tolvaptan, levosimendan, nesiritide, dopamine, dobutamine, and any kind of diuretics, regardless of the mode, dosage, and duration of administration. (3) Control individuals took either placebo, conventional treatment (PLC), or one of the drugs mentioned above. (4) Studies reported at least one of the following outcomes: (a) Serum creatinine (Cr); (b) Blood urea nitrogen (BUN); (c) Brain natriuretic peptide (BNP); (d) N-Terminal Pro-Brain Natriuretic Peptide (pro-BNP); (e) GFR; (f) Mortality (All-cause mortality or cardiovascular mortality within 60 days); (5) Randomized controlled trial (RCT) or retrospective study. (6) Studies published in English.

Exclusion criteria were as follows: (1) Unclear criteria for diagnosis and efficacy. (2) Animal studies, meta-analyses/reviews, conference abstracts, letters/responses to editors, guidelines, or case reports. (3) Incomplete data that could not be merged.

### 2.3 Search methods

PubMed, Embase, Cochrane Library, and Web of Science were searched independently by two researchers [Qianyu Lv (L.Q.Y.) and Qian Wu (W.Q)] up to March 2024, with no restrictions on document type, publication time, or publication status. The combination of Medical Subject Heading (MeSH) terms and their free words was used as the search strategy. MeSH terms included keywords, including “acute heart failure”, “acute decompensated heart failure”, “heart failure”, and recommended drugs: diuretics, vasopressin V2 receptor antagonists, ACEI, ARBs, natriuretic peptides administered alone, beta-blockers, Ivabradine, digitalis, levosimendan, phosphodiesterase III inhibitors, and SGLT2i.

### 2.4 Study selection

Two researchers (L.Q.Y. and W.Q) independently reviewed the titles, abstracts, and then the full texts of all searched articles. First of all, the possibly relevant research was imported into EndNote X9, and duplicates were removed automatically and manually. Then, titles and abstracts were reviewed to exclude unqualified literature, followed by full-text assessment. Any disagreement was resolved by discussion with a third researcher (LanLan.Li (L.L.L.)).

### 2.5 Data extraction and quality assessment

A Cochrane data extraction table was utilized to extract the following data: (1) General information of the article: title, first author, author's country, publication year; (2) Basic characteristics of the study population: age, sex, and included cases; (3) Details of the intervention: specific measures and intervention duration; (4) Levels of outcome indicators (including Cr, BUN, BNP, pro-BNP, GFR) before and after intervention; (5) Mortality rates. One researcher (L.Q.Y.) extracted data, while the other researcher (W.Q.) checked the data accuracy.

The study quality was independently assessed by two reviewers (L.Q.Y. and W.Q.) using the RoB 2 (22) in the following five bias domains: randomization process, allocation concealment, intervention blinding of participants and investigators, missing outcome data, outcome measurement and selection of reported results. An algorithm was employed to estimate the overall risk of bias, i.e., low risk, some concerns, or high risk. The above risk of bias assessment was conducted by two reviewers (L.Q.Y. and W.Q.), and any discrepancies were resolved by discussion with the third researcher (L.L.L.).

### 2.6 Statistical analysis

The statistical model based on the Bayesian framework was built using JAGS software 4.3.1 [gemtc package 0.8–2 and rjags package 4–10)] (Rstudio, Boston, MA, USA). Based on the clinical heterogeneity of the included trials (i.e., country, drug dose, and duration of administration), a random-effects model with four Markov chains was used for each outcome, with each chain producing 50,000 iterations (burn-in period of 20,000 iterations). The convergence of iterations was monitored using plots and the Gelman–Rubin–Brooks statistic (23). We also comparing the therapeutic effect of different drugs using a Surface Under the Cumulative Ranking curve (SUCRA), the closer SUCRA was to 100, the better the therapeutic effect. Model consistency was assessed using the deviation information criterion (DIC), with differences in DIC < 5 points indicating good consistency and consistency modeling was used (24). Heterogeneity was estimated using the *I*^2^ statistic with *I*^2^ values <25% indicating low heterogeneity, 25%–75%, moderate heterogeneity, and >75%, high heterogeneity (25). Publication bias was assessed by funnel plots. Network plots and funnel plots were drawn by Stata SE 15.0 (StataCorp, College Station, Texas, USA).

## 3 Results

### 3.1 Search outcomes

**Figure 1:**
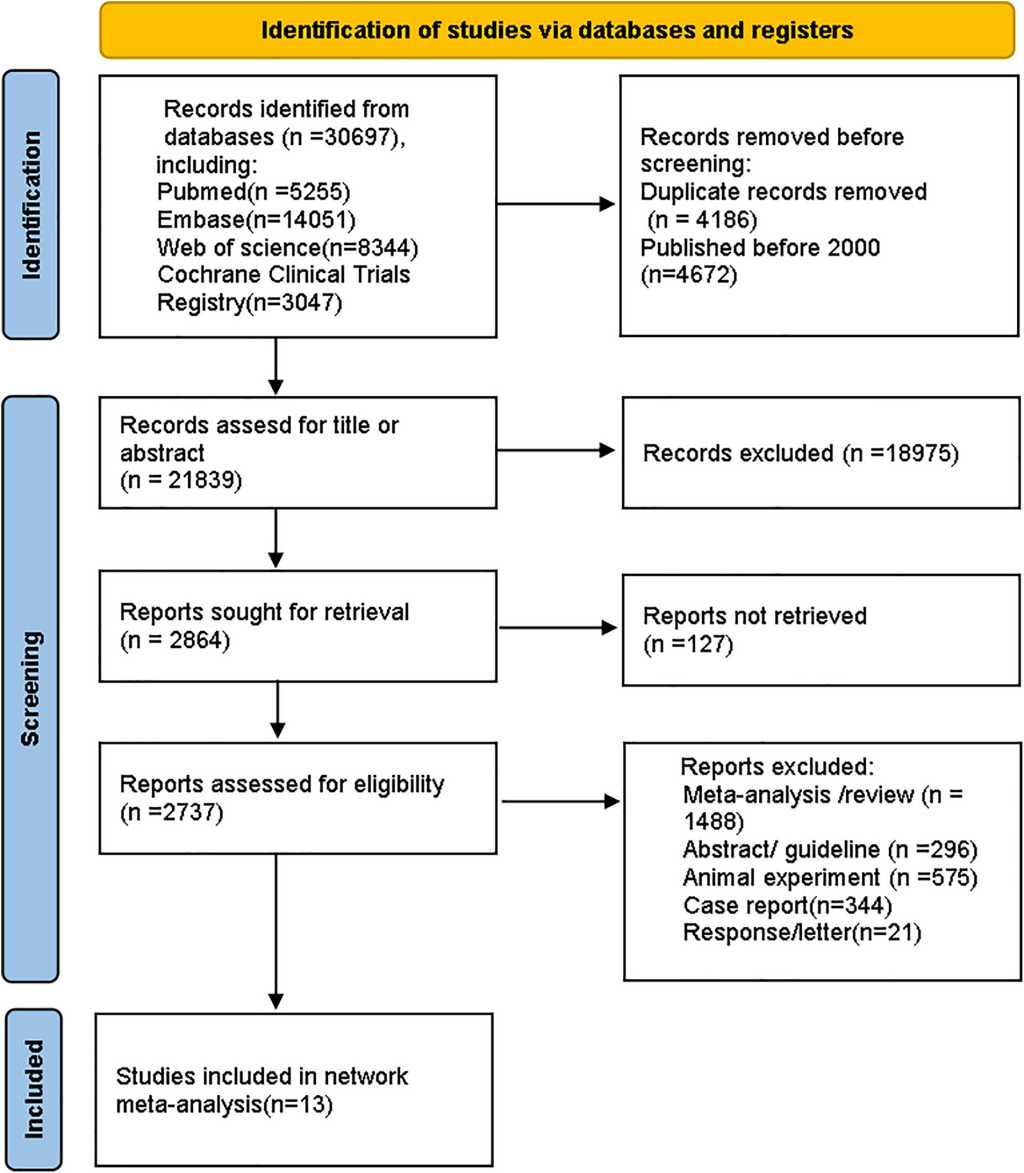
Flow diagram for study selection.

The process for selecting studies follows the updated PRISMA guidelines (26). A total of 30,697 records were identified from databases. After removing 4,186 duplicates and 4,672 studies that were published before 2,000, we reviewed the titles and abstracts of the remaining articles based on eligibility criteria. Ultimately, 13 studies were included. The screening process is shown in Figure 1.

### 3.2 Characteristics of included studies

Of the 13 eligible studies published from 2001 January to March 2024, 4 studies were conducted in North America (27–30), 3 in Europe (31–33), and 6 studies (34–39) in Asia. A total of 21,745 AHF patients with renal dysfunction were enrolled. The sample size of each article ranged from 21 to 759 participants, and the mean age ranged from 60.6 to79.4. Seven drugs (nesiritide, dopamine, tolvaptan, levosimendan, dobutamine, and high doses of diuretics (HDD, including spirolactone and furosemide) were included. Since 3 studies used high doses of furosemide and spironolactone (28, 31, 34), unlike conventional treatment, we combined the two drugs into an HDD group). As for study design, our NMA included 9 RCTs (27–32, 35, 37, 39), 1 prospective observational study (38), and 3 retrospective studies (33, 34, 36). All treatments were completed during hospitalization (Table 1).

**Table 1:** Main characteristics of the studies included in the network meta-analysis.

Author (year)	Study design	Sample size (*n*)		Age (years)		Sex (male%)		Intervention	Outcomes	Baseline NT-proBNP/BNP (pg/ml)	Baseline Cr (mg/dl)	Baseline GFR (ml/min/1.73 m^2^）
		Treatment	Control	Treatment	Control	Treatment	Control					
Matsue et al (38)	RCT	108	109	72.9 ± 8.9	72.9 ± 10.2	66.7	63.3	Oral tolvaptan 15 mg/day + conventional treatment for 2 days	Cr; BUN, GFR	849.4 ± 199.3(BNP)	1.43 ± 0.52	42.4 ± 12.4
								Conventional treatment for 2 days				
Uemura et al. (33)	retrospective study	33	36	75.1 ± 12.3	79.4 ± 9.9	45.5	52.8	Tolvaptan + loop diuretics	Cr, GFR; 180 d cardiovascular death	1,148.2 ± 1,621.5(BNP)	1.73 ± 0.9	27.2 ± 9.4
								High-dose loop diuretics (≥40 mg)				
Wan et al (26)	RCT	120	119	72.2 ± 3.6	70.8 ± 4.3	70.0	74.8	Dopamine 2 µg/kg per minute infusion for 72 h	Cr, NT-proBNP, 60 d all-cause death	5,452.3 ± 2,088.5 (NT-proBNP)	1.63 ± 0.11	44.36 ± 4.19
								Placebo				
Wan et al (26)	RCT	119	119	69.3 ± 5.6	70.8 ± 4.3	76.4	74.8	Nesiritide 0.005 µg/kg per minute infusion for 72 h	Cr, NT-proBNP, 60 d all-cause death	5,452.3 ± 2,088.5 (NT-proBNP)	1.63 ± 0.11	44.36 ± 4.19
								Placebo				
Rafouli et al (32)	open-label observational study	48	48	—	—	—	—	Levosimendan	Cr, BNP	—	—	—
								Conventional treatment				
Tamaki et al (34)	RCT	26	24	79.0 ± 7.0	75.0 ± 10.0	54.0	46.0	Tolvaptan was started from an initial dose of 7.5 mg/day, maximum dose was set at 15.0 mg/day	Cr; BUN; GFR; BNP	652.7 ± 564.9(BNP)	1.41 ± 1.26	45.85 ± 18.49
								Conventional treatment				
Greene et al. (27)	RCT	82	178	—	—	—	—	100 mg spironolactone daily	NT-proBNP; 60 d all-cause death	6,216.6 ± 1,985.9(NT-proBNP)	—	43.94 ± 1.76
								Conventional treatment				
Ono et al. (35)	retrospective observational study	31	27	76.0 ± 14.2	78.4 ± 9.5	45.0	44.0	Continuous treatment with tolvaptan for 6 months or more	GFR, BNP, 180 d cardiovascular death	1,079.8 ± 874.2(BNP)	1.58 ± 0.7	35.0 ± 13.6
								Conventional treatment				
John et al. (36)	RCT	22	36	60.6 ± 11.0	65.1 ± 12.7	77.2	77.8	Levosimendan on every even day as a loading dose of 12 mcg/kg over 10 min followed by a 0.1 mcg/kg/min infusion, administered for 24 h	Cr; GFR	—	2.88 ± 1.61	40.14 ± 14.17
								dobutamine on odd days as infusion of 5 mcg/kg/min				
Giamouzis et al. (30)	RCT	30	30	74.1 ± 11.7	77.4 ± 10.7	50.0	28.0	Dopamine 5 *μ*g k g−1 min−1 continuous infusion for 8 h + conventional treatment	Cr; GFR; 60 d all-cause death	1,462 ± 1,082.3(BNP)	1.22 ± 0.38	58.2 ± 17.8
								High dose of furosemide 20 mg/h continuous infusion for 8 h + conventional treatment				
Vaduganathan et al. (29)	RCT	386	373	65.4 ± 12.1	66.1 ± 12.7	18.9	18.5	Tolvaptan 30 mg/day	Cr; GFR; BUN;	8,448.2 ± 3,088.5(NT-proBNP)	1.57 ± 0.53	51.39 ± 19.08
								Placebo				
Chen et al. (28)	RCT	122	119	71.0 ± 3.3	71.8 ± 3.5	69.0	72.0	Dopamine (2 ug/kg/min) for 72 h infused via local guideline stipulated vascular access	Cr; NT-proBNP; 60 d all-cause death	4,987.2 ± 1,743.6(NT-proBNP)	1.64 ± 0.13	43.1 ± 5.7
								Placebo				
Chen et al. (28)	RCT	119	119	70.0 ± 3.1	67.4 ± 3.2	75.0	78.0	Nesiritide(0.005 *μ*g/kg/min) for 72 h infused via peripheral intravenous access	Cr; NT-proBNP; 60 d all-cause death	4,987.2 ± 1,743.6(NT-proBNP)	1.64 ± 0.13	43.1 ± 5.7
								Placebo				
Matsue et al. (37)	prospective observational study	44	70	72.2 ± 11.8	70.8 ± 10.5	51.4	22.2	Tolvaptan 15 mg per day;	BNP	926.3 ± 571.4(BNP)	1.62 ± 0.83	42.1 ± 24.2
								conventional treatment				
Fedele et al. (31)	RCT	14	7	71.8 ± 9.0	77.4 ± 7.2	78.6	100	Levosimendan,10 min i.v. loading dose (6 *μ*g/kg) followed by an infusion (0.1 *μ*g/kg/min) for 24 h	Cr; GFR; BUN	—	1.73 ± 0.33	40.25 ± 8.0

### 3.3 Quality assessment

**Figure 2:**
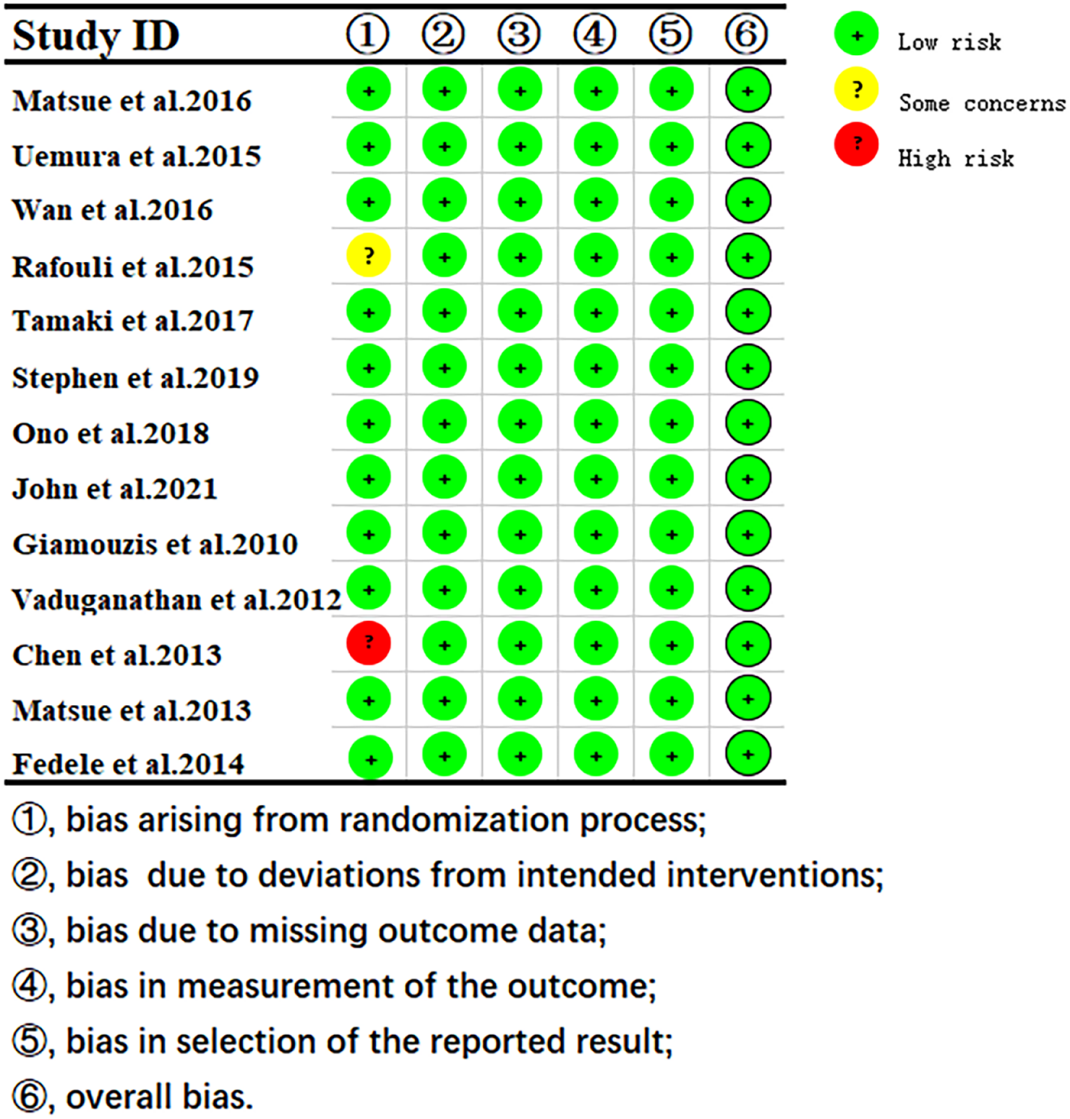
Risk of bias of RoB 2 assessment using robvis.

The quality assessment results showed that 1 study had some concerns about publication bias, and others showed a low risk of bias. (Figure 2).

### 3.4 NMA

#### 3.4.1 Cardiac function indicator

##### 3.4.1.1 NT-proBNP (pg/ml)

**Figure 3:**
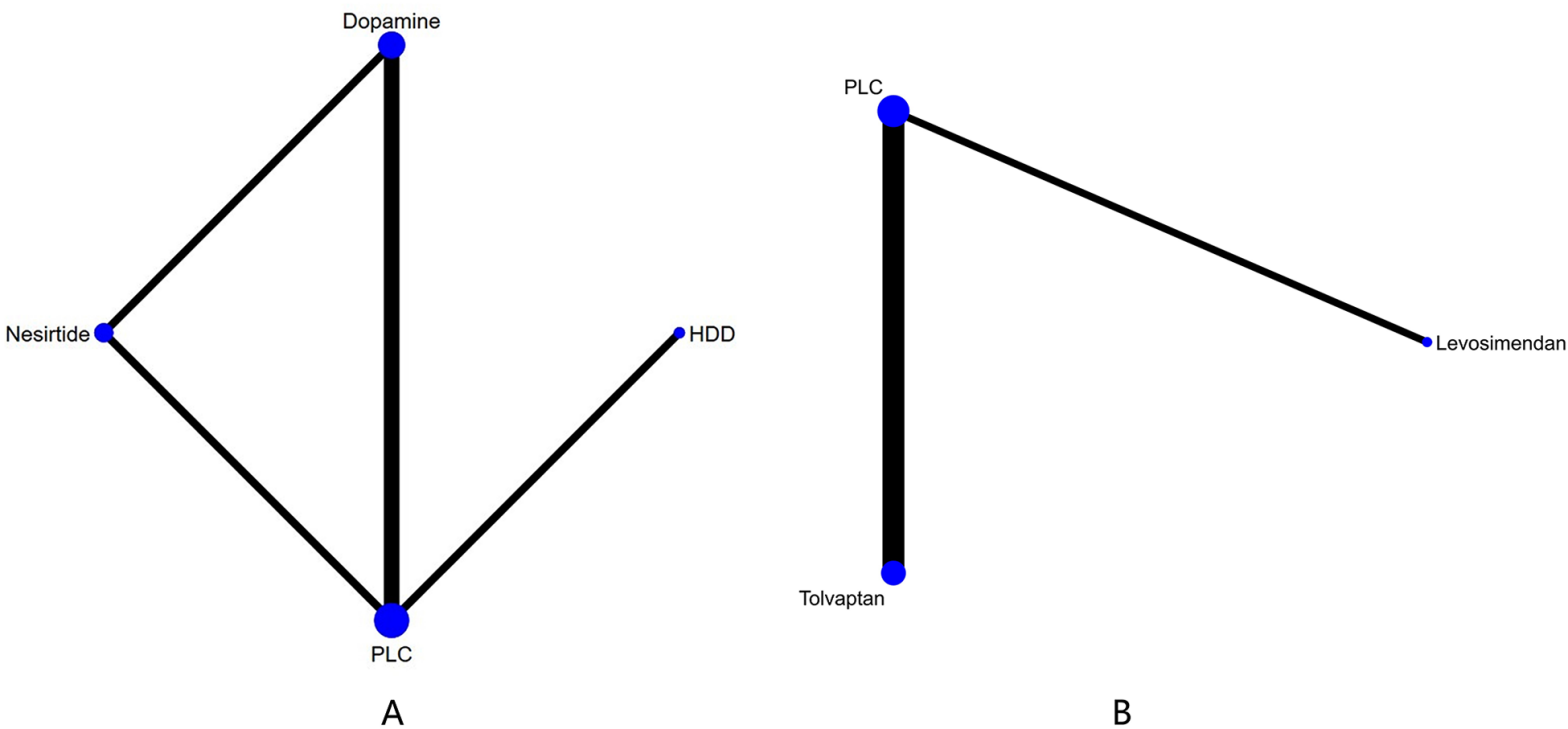
Network plots of cardiac function indexes. **(A)** Network plot for NT-proBNP. **(B)** Network plot for BNP. PLC stands for receiving either placebo treatment or conventional treatment. Nodes stand for the comparison between treatments and the size is proportional to the number of subjects. The width of the lines is proportional to the number of trials per pair of interventions.

Three studies (27–29) compared the effect of drugs (diuretics, dopamine, and nesiritide) on NT-proBNP (Figure 3A, Table 2). DIC comparison results showed good agreement (DIC, 13.30 vs. 13.32). According to the NMA results, HDD was the most likely to be the optimal drug for reducing NT-proBNP levels, followed by dopamine, and nesiritide (SUCRA: HDD, 92%, dopamine, 67%, nesiritide, 34%). Among them, the pairwise comparison indicated that HDD significantly reduced NT-proBNP levels compared to PLC [MD = −950.24, 95% CrI (−1,832.21, −64.12)]. Besides, HDD reduced NT-proBNP levels compared to dopamine [MD = −342.31, 95% CrI (−1,424.03, 734.56)] and nesiritide [MD = −697.91, 95% CrI (−1,906.77, 498.05)], with no significant difference.

**Table 2:** Relative effects of different drugs on NT-proBNP (reported as odds ratios (OR) or weighted mean differences (MD) with 95% credible intervals (CrI).

HDD			
−342.31 (−1424.03, 734.56)	Dopamine		
−697.91 (−1,906.77, 498.05)	−355.06 (−1,183.4, 463.74)	Nesiritide	
**−950.24** **(−1,832.21, −64.12)**	−607.28 (−1,236.6, 18.96)	−252.55 (−1,069.23, 568)	PLC

PLC stands for receiving either placebo treatment or conventional treatment. Estimates are presented as MD and 95% CrI. Comparisons between treatments should be read from left to right and the comparison result is presented at the intersection of the column-defining drug and the row-defining drug. Significant results are exhibited in bold.

##### 3.4.1.2 BNP (pg/ml)

In total, four studies (33, 35, 36, 38) evaluated the effect of levosimendan and tolvaptan on BNP levels in AHF patients with renal dysfunction (Figure 3B). The main findings are shown in Table 3. DIC comparison results showed good agreement (15.34 vs. 15.36). Levosimendan showed the best efficacy in reducing BNP levels, followed by tolvaptan (SUCRA, levosimendan, 95%, tolvaptan, 28%). However, there were no significant differences among levosimendan, tolvaptan, and PLC.

**Table 3:** Relative effects of different drugs on BNP (reported as OR or weighted MD with 95% CrI).

Levosimendan		
−505.27 (−1,072.03, 66.76)	PLC	
−504.44 (−1,165.19, 137.34)	4.84 (−334.37, 306.3)	Tolvaptan

PLC stands for receiving either placebo treatment or conventional treatment. Estimates are presented as MD and 95% CrI. The estimate of effectiveness is presented at the intersection of the column-defining drug and the row-defining drug.

#### 3.4.2 Renal function indicators

##### 3.4.2.1 GFR (ml/min 1.73 m^2^)

**Figure 4:**
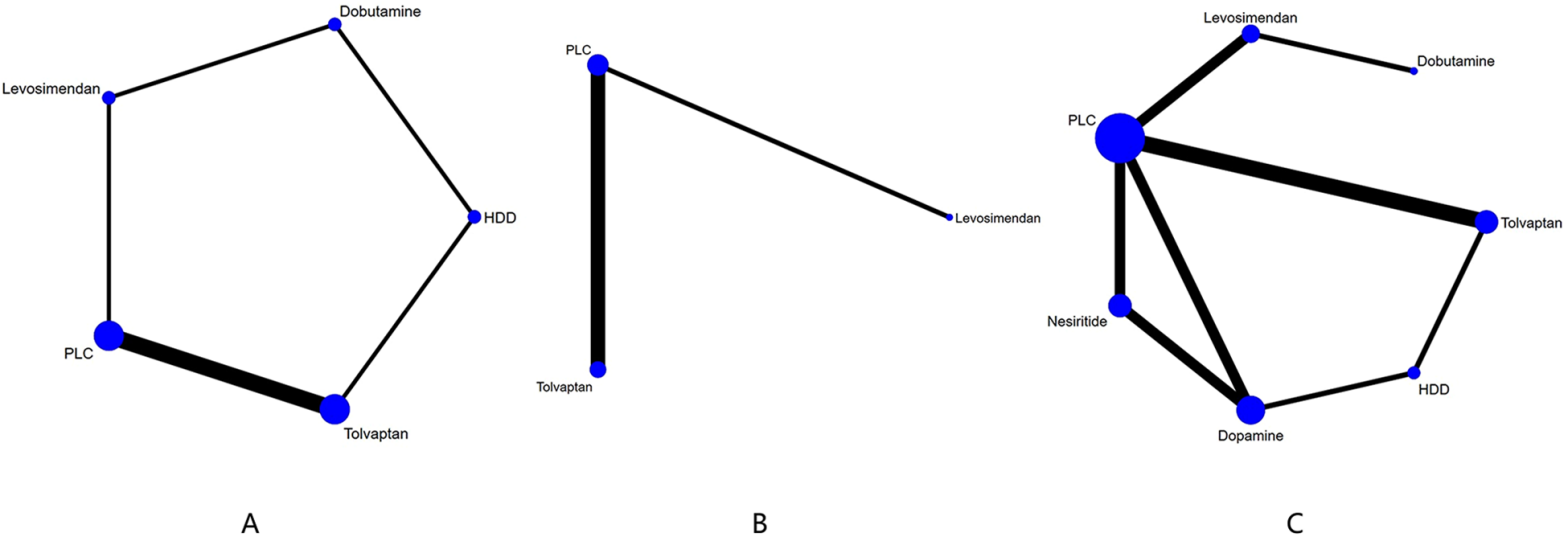
Network plot and results of NMA. **(A)** Network plot for GFR). **(B)** Network plot for BUN. **(C)** Network plot for Cr. PLC stands for receiving either placebo treatment or conventional treatment. Nodes stand for the comparison between treatments and the size is proportional to the number of participants. The width of the lines is proportional to the number of trials in each pair of interventions.

Five drugs across 8 studies (30–32, 34–37, 39) were analyzed to compare their effects on GFR levels (Figure 4A, Table 4). Comparison of the consistency model and the inconsistency model results showed good agreement (31.83 vs. 32.19). Levosimendan tended to be the most effective drug in increasing GFR levels, followed by dobutamine, tolvaptan, and HDD (SUCRA: levosimendan, 98%, dobutamine, 68%, tolvaptan, 38%, PLC, 31%, HDD, 14%). Levosimendan, dobutamine, and tolvaptan may have more positive effects than PLC, while HDD may have a more negative effect than PLC. Levosimendan significantly increased GFR levels compared to PLC [MD = 14.46; 95% CrI (3.88, 25.97)] and tolvaptan [MD = 13.83; 95% CrI (2.31, 25.33)].

**Table 4:** Relative effects of different drugs on GFR (reported as OR or weighted MD with 95% CrI).

Dobutamine				
8.93 (−2.97, 19.46)	HDD			
−7.82 (−18.2, 1.99)	−16.76 (−28.8, −3.87)	Levosimendan		
6.6 (−5.45, 19.25)	−2.42 (−11.73, 9.35)	**14.46 (3.88, 25.97)**	PLC	
6 (−6.65, 18.11)	−2.93 (−12.21, 7.36)	**13.83 (2.31, 25.33)**	−0.45 (−6.52, 3.95)	Tolvaptan

PLC stands for receiving either placebo treatment or conventional treatment. Estimates are displayed as MD and 95% CrI. The estimate of effectiveness is presented at the intersection of the column-defining drug and the row-defining drug. Significant results are exhibited in bold.

##### 3.4.2.2 BUN (mg/dl)

The network plot of BUN is shown in Figure 4B, including 2 drugs (levosimendan, tolvaptan) from 4 studies (30, 32, 35, 39), and the main finding is shown in Table 5. DIC comparison results showed good agreement (17.01 vs. 16.81). Compared with placebo, both levosimendan and tolvaptan reduced BUN levels in patients. Additionally, levosimendan ranked first, followed by tolvaptan and placebo (SUCRA: levosimendan, 93%, tolvaptan, 41%, PLC, 14%), but the difference in the comparison between any two drugs was not significant.

**Table 5:** Relative effects of different drugs on BUN (reported as OR or weighted MD with 95% CrI).

Levosimendan		
−14.58 (−32.78, 3.76)	PLC	
−13.19 (−32.39, 6.69)	1.12 (−5.42, 9.17)	Tolvaptan

PLC stands for receiving either placebo treatment or conventional treatment. Estimates are displayed as MD and 95% CrI. The estimate of drug effectiveness is presented at the intersection of the column-defining drug and the row-defining drug.

##### 3.4.2.3 Cr (mg/dl)

Ten studies (27, 29–35, 37, 39) analyzed the changes in Cr levels, with seven drugs included (Figure 4C, Table 6). DIC comparison results showed good agreement (45.33 vs. 46.01). NMA showed that each drug tended to reduce Cr compared with PLC. Although the differences of pairwise comparisons were not substantial, levosimendan was the most promising drug for reducing Cr levels, followed by dobutamine, dopamine, nesiritide, tolvaptan, and HDD (SUCRA: levosimendan 80%, dobutamine 67%, dopamine, 58%, nesiritide, 45%, PLC, 38%, tolvaptan, 33%, HDD, 26%). Among them, levosimendan, dobutamine, dopamine, and nesiritide may have better efficacy than PLC in reducing Cr levels, while tolvaptan and HDD may have worse efficacy than PLC.

**Table 6:** Relative effects of different drugs on Cr (reported as OR or weighted MD with 95% CrI).

Dobutamine						
−0.15 (−0.89, 0.59)	Dopamine					
−0.25 (−1.02, 0.54)	−0.1 (−0.37, 0.18)	HDD				
−0.05 (−0.72, 0.65)	0.11 (−0.17, 0.4)	0.21 (−0.17, 0.58)	Levosimendan			
−0.18 (−0.92, 0.56)	−0.02 (−0.21, 0.15)	0.08 (−0.25, 0.37)	−0.13 (−0.43, 0.14)	Nesiritide		
−0.19 (−0.91, 0.53)	−0.03 (−0.22, 0.12)	0.06 (−0.25, 0.34)	−0.14 (−0.39, 0.07)	−0.01 (−0.2, 0.16)	PLC	
−0.2 (−0.94, 0.53)	−0.05 (−0.27, 0.16)	0.05 (−0.26, 0.34)	−0.16 (−0.44, 0.11)	−0.03 (−0.25, 0.2)	−0.02 (−0.16, 0.15)	Tolvaptan

PLC stands for receiving either placebo treatment or conventional treatment. Estimates are displayed as MD and 95% CrI. The drug effectiveness is presented at the intersection of the column-defining drug and the row-defining drug.

#### 3.4.3 Mortality

##### 3.4.3.1 60-day all-cause mortality

**Figure 5:**
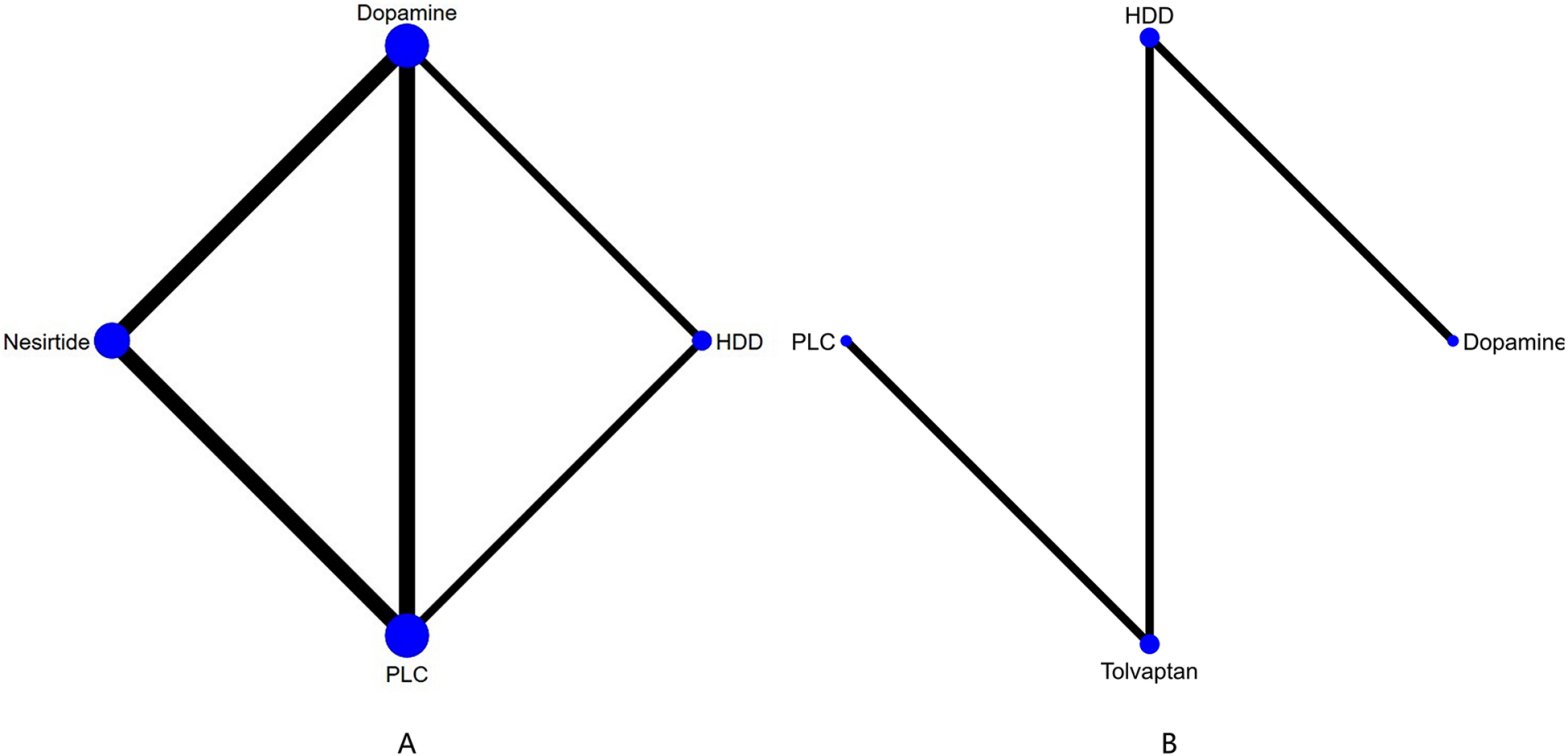
Network plot and results of NMA. **(A)** Network plot for 60-day all-cause mortality. **(B)** Network plot for 60-day cardiac-cause mortality. PLC stands for receiving either placebo treatment or conventional treatment. Nodes represent the comparison between treatments and the size is proportional to the number of subjects. The width of the lines is proportional to the number of trials in each pair of interventions.

In 60-day all-cause mortality, 4 trials (27–29, 31) with 3 drugs (HDD, nersirtide, dopamine) were involved (Figure 5A, Table 7). Comparison of the consistency model and the inconsistency model results showed good agreement (14.93 vs. 16.94). Pairwise comparisons did not show notable differences in the rate of 60-day all-cause mortality. Nesiritide was associated with the lowest rate of all-cause mortality within 60 days, followed by HDD and dopamine (SUCRA: nesiritide 75%, HDD, 55%, dopamine, 39%, PLC, 29%), all of which were safer than placebo.

**Table 7:** Relative effects of different drugs on all-cause mortality within 60 days (reported as OR or weighted MD with 95% CrI).

HDD			
0.91 (0.34, 2.33)	Dopamine		
1.21 (0.42, 3.51)	1.33 (0.67, 2.74)	Nesirtide	
0.83 (0.35, 1.95)	0.92 (0.5, 1.71)	0.69 (0.34, 1.37)	PLC

PLC stands for receiving either placebo treatment or conventional treatment. Estimates are displayed as MD and 95% (CrI. The drug effectiveness is presented at the intersection of the column-defining drug and the row-defining drug.

##### 3.4.3.2 Cardiovascular death

Three studies (31, 34, 36) analyzed the effect of different drugs on cardiovascular mortality (Figure 5B, Table 8). DIC comparison results showed good agreement (4.51 vs. 8.71). The pairwise comparisons did not show marked differences. Tolvaptan, dopamine, and HDD had the lowest to highest rate of cardiovascular death (SUCRA: tolvaptan 77%, dopamine, 50%, PLC, 38%, HDD, 33%). Among them, the effect of HDD on cardiovascular death within 60 days may be inferior to that of PLC, while the effect of dopamine and tolvaptan may be superior to that of PLC.

**Table 8:** Relative effects of different drugs on cardiovascular mortality within 60 days (reported as point estimates of OR or weighted MD with 95% CrI).

Dopamine			
0.63 (0.06, 5.41)	HDD		
0.06 (0, 57,259,656.86)	0.09 (0, 82,79,2633.06)	PLC	
3.21 (0.11, 180.84)	4.89 (0.47, 176.29)	60.17 (0, 41,077,393,324,309)	Tolvaptan

PLC stands for receiving either placebo treatment or conventional treatment. Estimates are displayed as MD and 95% CrI. The estimate of drug effectiveness is presented at the intersection of the column-defining drug and the row-defining drug.

### 3.5 Consistency

The consistency model and inconsistency model were compared using DIC, with changes in DIC < 5 for all closed-loop models indicating good consistency.

## 4 Discussion

To date, the management of AHF with renal dysfunction remains challenging, and evidence is lacking in many areas. To analyze the efficacy of current therapies on cardiac function, renal function and clinical outcomes in patients with AHF with renal dysfunction, we performed this NMA. Our study is the first comparison of various drugs with either placebo or conventional treatment. Since conventional treatment mostly uses low-dose diuretics, we enrolled studies using high-dose diuretics, regardless of the type (furosemide and spironolactone here) as the High-Dose Diuretic group and compared with other drugs and PLC.

In terms of cardiac function indicators, NMA results indicated that diuretics had better effects than dopamine and nesiritide on reducing NT-proBNP, with a significant difference compared with PLC. As for BNP, the effect of levosimendan appeared to be the best, with no significant difference compared with PLC. NT-proBNP, released by cardiomyocytes against ventricular stress, can effectively regulate blood pressure and electrolyte balance, with strong associations with ventricular hypertrophy and systolic dysfunction (40, 41). NT-proBNP level can effectively evaluate the clinical efficacy in HF patients (42), and is also an independent risk factor for sudden cardiac death (41, 43). In addition, since NT-proBNP is excreted through glomerular filtration and is easily affected by renal function (44), it is therefore an important indicator for patients with AHF and renal dysfunction. Based on our NMA, HDD can significantly reduce NT-proBNP levels. The conventional treatment for AHF is diuretics, however, there are ongoing debates on the therapeutic effectiveness of high-dose diuretics in patients with AHF and renal dysfunction. Furosemide is a loop diuretic while spironolactone is an aldosterone antagonist. Some studies have found that high-dose diuretics are more effective in increasing urine volume in AHF patients than low-dose diuretics (45, 46). Therefore, high-dose diuretics may effectively reduce water and sodium retention, and alleviate cardiac preload, ventricular wall stress, and then, the secretion of NT-pro-BNP in cardiomyocytes is reduced.

BNP is a cardiac neuroendocrine hormone released by ventricular muscles during myocardial damage and has great value in the early prediction of death and other adverse events in AHF patients (47). One study showed that lower BNP levels at discharge were associated with lower mortality (48), and the WRF with higher BNP levels at discharge was associated with poor prognosis. Therefore, determining which drugs help reduce BNP levels has great prognostic implications. Although our NMA results showed that no included drugs significantly reduced BNP levels, levosimendan seems more effective than other drugs. In contrast to our findings, a meta-analysis demonstrated that levosimendan improved cardiac function and significantly reduced plasma BNP levels in patients with decompensated HF (49). These differences may be due to heterogeneity in the study population, including differences in baseline cardiac and renal function and therefore these results still warrant consideration. Levosimendan, a calcium sensitizer, is a novel positive inotropic drug (32) that specifically binds to troponin C in cardiomyocytes to enhance muscle strength without increasing myocardial oxygen consumption, and thus, has better safety (50, 51). The main mechanism of levosimendan in reducing BNP concentration is to enhance myocardial contractility, dilate coronary artery, and reduce cardiac load, ventricular wall stress, and BNP release by ventricular myocytes, without increasing myocardial oxygen consumption (52). Additionally, it inhibits the production of tumor necrosis factor-α, interleukin-6, leukocytin-8, and BNP (53).

As for renal function, levosimendan significantly increased GFR values. Although there was no significant difference in lowering BUN and Cr, levosimendan also showed better protective effects on the kidneys than other drugs. Similar to our findings, a meta-analysis of 529 patients suggested that levosimendan may reduce the incidence of perioperative acute kidney injury (54) by increasing renal blood flow through renal vasodilation (55). Levosimendan has been shown to increase GFR and protect the kidney in patients with cardiorenal syndrome (56–58). The mechanism may also include activation of renal K^+^ ATP channels in the bulbar arterioles, resulting in shortened duration of action potential in cardiomyocytes, hyperpolarization of vascular smooth muscle cells, reduced calcium flow, vasodilation, and increased blood perfusion (59). Furthermore, it can completely block angiotensinⅡ-induced mesangial cell contraction, thereby increasing the surface area of glomerular capillaries and protecting against acute renal failure (60, 61). In addition, other potential mechanisms of levosimendan involve anti-inflammatory and anti-apoptotic effects (62, 63). Mullens et al. (64) suggested that higher central venous pressure was related to higher renal venous pressure and WRF to a certain extent, resulting in decreased GFR and renal insufficiency. Levosimendan can improve renal function by reducing central venous pressure through right ventricular function. Notably, HDD may be inferior to placebo in improving GFR and Cr. Previous studies have shown that diuretics are an important method to reduce fluid retention in patients with acutely decompensated HF (ADHF), but they may lead to WRF (65–67). These results suggest that HDD should be used with caution in patients with AHF and renal insufficiency. Our results showed that tolvaptan may have a protective effect on kidney function, although there was no statistical difference. As a novel diuretic, tolvaptan increases urine output by inhibiting vasopressin receptors in the renal tubules. In a randomized controlled meta-analysis, tolvaptan significantly improved dyspnea symptoms compared with controls, but had little effect on kidney function (68). We hypothesize that tolvaptan may be beneficial in improving acute decompensated HF (ADHF) with renal insufficiency without reducing renal function, but large-scale clinical trials are still needed to confirm this. Besides, a new diuretic SGLT-2i has attracted our attention because of its potential applicability in AHF (69). SGLT-2i is believed to have some renal protection in the treatment of HF (70). A meta-analysis also showed that it can improve cardiovascular and renal outcomes in patients with chronic kidney disease (71). However, our search in the database did not find any clinical studies of SGLT-2i in the treatment of AHF with renal insufficiency, which is contrary to expectation. Therefore, we look forward to the clinical application of SGLT-2i in this field.

At last, we assessed the mortality of various drugs. There was no significant difference in the effect of included drugs on 60-day all-cause mortality and cardiovascular mortality. In terms of all-cause mortality, the efficacy of HDD, nersirtide, and dopamine was compared, and nesiritide had the best effect. Nesiritide is a recombinant B-type natriuretic peptide with vasodilatory effects (15, 72). Several meta-analyses have reported the effect of nesiritide on the mortality of AHF patients. Sackner Bernstein et al. (73) found that nesiritide may be associated with a higher risk of death after acute decompensated heart failure (ADHF) treatment, while Abraham (74) and Bin Yan (75) showed that nesiritide did not increase mortality of ADHF patients after short-term and long-term treatment. Another meta-analysis evaluated the effects of nesiritide on ADHF patients and found that high doses of nesiritide may increase the risk of WRF, however, standard and low doses of nesiritide may not affect renal function (76). Clinical trials of nesiritide in AHF with renal dysfunction are limited, and more studies are needed to validate its efficacy. In terms of reducing cardiovascular mortality, three regimens, including HDD, nesiritide, dopamine, and tolvaptan, were included, of which tolvaptan had the best effect. However, due to the small number of included studies and inconsistent follow-up time [2 studies reported cardiovascular death during 180 days (34, 36), and 1 study (31) reported 60 days], the results still need validation.

## 5 Strengths and limitations

To date, this is the first NMA to compare and rank the efficacy of different drugs on cardiac function, renal function, and clinical outcomes in patients with AHF with renal insufficiency. This NMA provides valuable references on the optimal drug for patients with AHF and renal insufficiency. The study did have some limitations, though. First, the limited studies and small sample size may impact the accuracy and applicability of the obtained results. Second, there were differences in the dosage, duration, and mode of administration across the included studies, which may lead to heterogeneity in the studies. This review focuses heavily on biochemical markers and lacks descriptions of some symptom improvements, which limits the practical application of the findings. In addition, newer therapies such as angiotensin receptor neprilysin inhibitors and SGLT2i are not included in this article, and we look forward to more relevant studies in the future.Finally, subgroup analysis was not performed due to the limited number of studies,reduces the specificity of the conclusions.

Therefore, more high-quality RCTs are needed to validate our findings.

## 6 Conclusions

We used a Bayesian NMA to analyze the advantages and disadvantages of nesiritide, dopamine, tolvaptan, levosimendan, dobutamine, and HDD (including furosemide and spirolactone) in the treatment of patients with AHF and renal insufficiency. However, no single drug can optimally improve all indicators in these patients. HDD had a significant effect in reducing NT-proBNP levels. No drug has a significant difference in reducing BNP. Levosimendan was the best at improving GFR levels, although the difference was not significant, it seems also to be better at reducing BUN and Cr levels. In terms of clinical outcomes, the rates of 60-day all-cause death and cardiovascular death were all similar across drugs. Due to limitations in clinical trials, future trials with larger sample sizes, longer follow-up period, and more rigorous design are warranted to confirm these findings.

## Data Availability

The original contributions presented in the study are included in the article/Supplementary Material, further inquiries can be directed to the corresponding author.
